# The Combined Effects of Precision-Controlled Temperature and Relative Humidity on Artificial Ripening and Quality of Date Fruit

**DOI:** 10.3390/foods10112636

**Published:** 2021-10-30

**Authors:** Maged Mohammed, Abdelkader Sallam, Nashi Alqahtani, Muhammad Munir

**Affiliations:** 1 Date Palm Research Center of Excellence, King Faisal University, Al-Ahsa 31982, Saudi Arabia; nalqahtani@kfu.edu.sa (N.A.); mmunir@kfu.edu.sa (M.M.); 2 Agricultural and Biosystems Engineering Department, Faculty of Agriculture, Menoufia University, Shebin El Koum 32514, Egypt; 3 Plant Production Department, College of Technology and Development, Zagazig University, Zagazig 44519, Egypt; aasallam@yahoo.com; 4 Department of Food and Nutrition Sciences, College of Agricultural and Food Sciences, King Faisal University, Al-Ahsa 31982, Saudi Arabia

**Keywords:** date palm, Biser, postharvest, modified RH, real-time data logging, sensors, physicochemical, weight loss

## Abstract

Due to climatic variation, in-situ date palm fruit ripening is significantly delayed, and some fruits (Biser) cannot become ripe naturally on the tree. Because of that issue, the vast quantity of produce is mere wasted. Few traditional methods are adopted to ripe these unripe fruits through open sun drying or solar tunnel dehydration techniques. However, these methods have minimal use due to ambient temperature and relative humidity (RH) instability. Therefore, the present study was designed to find a precise combination of temperature and RH to artificially ripe the unripe Biser fruits under controlled environment chambers. For that purpose, eighteen automated artificial ripening systems were developed. The Biser fruits (cv. Khalas) were placed immediately after harvesting in the treatment chambers of the systems with three set-point temperatures (45, 50, and 55 °C) and six set-point RH (30, 35, 40, 45, 50, and 55%) until ripening. The optimal treatment combination for artificial ripening of Biser fruits was 50 °C and 50% RH. This combination provided good fruit size, color, firmness, total soluble solids (TSS), pH, and sugars content. As a result, there was a reduction in fruit weight loss and had optimum fruit ripening time. On the other hand, low temperature and RH delayed the ripening process, deteriorated fruit quality, and caused more weight loss. Although the combination of the highest temperature and RH (55 °C and 55%) reduced ripening time, the fruits have higher weight loss and negative quality. Therefore, the artificial ripening of unripe date palm Biser fruits can be achieved using 50 °C temperature and 50% RH combination. These findings can be applied in the field using solar energy systems on a commercial scale to reduce the postharvest loss of date palm fruits.

## 1. Introduction

The cultivation of the date palm contributes to eradicating food malnutrition and insecurity in countries where it is grown. Date palm fruit is a good source of high nutritious value food that can be easily stored for a long time [1]. Date fruits on the bunch or entire bunches on a date palm tree do not equally ripen simultaneously. Several pickings may have to be made over several weeks [2]. Fruit ripening is a process that causes them to become more palatable. In general, the fruit becomes sweeter, less green or attain specific cultivar color, and softer as it ripens [3]. Depending on the genotype, cultural practices, environment, season, and amount of applied irrigation water, the date fruit exhibits a high diversity in shape, texture, chemical composition, and color [4]. The sufficient applied irrigation water substantially enhances date fruit quality and reduces unripe fruit (Biser) percentage [5,6]. Harvesting mature date palm fruits at the right time is critical for growers to get the most out of their investment. For the best quantity and quality of dates, choosing the best fruit harvesting time is vital [7]. The time is taken to fruit ripening of date palm varied with the cultivar. However, knowledge about fruit ripening processes is crucial. To determine the degree of ripeness in a fruit, ripening indices based on the most significant changes in physical and chemical characteristics that occur during the ripening process are essential [8,9,10,11,12].

A phase change in fruit development is triggered by the stimulation of the ripening process, which is aided by a significant change in primary and secondary metabolism. Carotenoids, flavor and volatile compounds, antioxidants, and sugars are formed as a result of these coordinated changes, all of which improve the nutritional quality of the fruits [13]. The ripening responses are triggered by enzymes. The color of the fruit skin changes from green to red, yellow, or blue as chlorophyll is degraded and new pigments are formed. The taste of fruit changes from sour to neutral due to the breakdown of acids. The amylase enzyme breaks down starch into sugar, which makes fruit sweeter [14,15,16].

Except for cultivar Barhi, which may follow an ethylene-independent route [17], date palms are classified as climacteric fruit. The Khalal stage begins after fruit set and the fruit is green in color, Biser stage starts when the fruit is yellow, Rutab stage begins when a soft, brown area develops primarily on the bottom end of the fruit, and Tamar stage occurs when the entire fruit becomes soft, brown, and juicy [18]. In July-August, the peak dates production time in Saudi Arabia, the ripening season of dates begins with an increase in summer temperatures and a decrease in RH. Unfortunately, in recent years, the ripening of date fruit within the spathe has been inconsistent in many cultivars. In certain cases, the fruits never developed from Biser to Rutab or Tamar. In the case of late-maturing cultivars, the fruit losses are much greater. At this stage of growth, the developmental fruits are vulnerable to insect pests and diseases that attack them rapidly. The pathogens feed on these fruits and reproduce, causing additional damage to future crops [19]. Some date palm cultivars, such as Barhi, Helali, and Sukkary, are also consumed fresh in the Biser stage. As a result, fresh fruits are oversupplied in the local market, and excessive production is usually wasted. This waste cannot be reduced due to a lack of on-farm cold storage, shipping, and preservation technology [20,21].

Moreover, the majority of farmers harvest the unripe date fruits and feed them to their livestock. Due to climate variability, date fruits at the Rutab stage might sometimes take a long time to reach the Tamar stage. Therefore, date palm growers frequently use artificial ripening chemicals to speed up the ripening process [22]. The use of sodium chloride appeared to be more effective in a trial on unripe Dhakki dates, resulting in a 75% increase in ripening [23]. Similarly, the application of 2% brine solution increased the artificial ripening of unripe Khalal Dakkai dates by up to 75% [19].

However, in recent years, the effect of artificial ripening using chemicals has become questionable because of various health-related issues. Therefore, alternative fruit ripening techniques gained interest to ripe the unripe date-fruits, which are simple, environment friendly, and cost-effective. When ripening is not completed on the palm or early rains threaten to damage the crop, artificial heat treatment, not exceeding 50 °C, can be used to ripe date palm fruits (cv. Deglet Noor) [24,25,26]. An artificially ripening study on unripe Khalal and Rutab fruits of the date palm cv. Khuneizi revealed that a treatment combination of freezing (−8 °C) followed by placing fruits at 50 °C for 48 h was the most effective [27]. Similarly, microwave treatment at 480 W for 50 s shortened the ripening time of unripe Khalal date fruits of cv. Dhakki from 288 h to 40 h [28]. To convert Rutab stage dates into table dates, Haider et al. [29] used oven-drying and solar dehydration techniques. The oven-drying ripening approach outperformed the traditional sun-drying method in terms of physicochemical and sensory characteristics. During the storage of dates, pests are effectively controlled by modifying the atmosphere with CO_2_ during storage and using solar-powered heating systems after harvesting [24,25,26]. Similarly, in modern packing houses, prematurely harvested dates are ripened in controlled environments with varying degrees of temperature and humidity depending on the cultivar [30]. Keeping in view the importance of alternative methods of artificial fruit ripening, the present study was conducted to investigate the effects of precise controlled temperatures and RH combination on artificial ripening time, weight loss, and some physicochemical characteristics of date fruit (cv. Khalas) at the Biser stage.

## 2. Materials and Methods

### 2.1. Plant Material and Sample Collection Site

**Figure 1:**
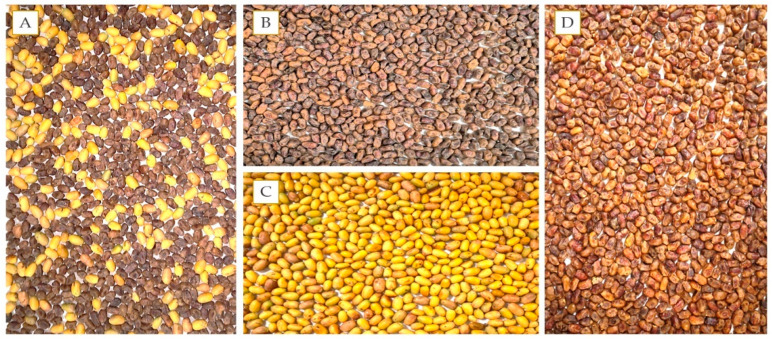
(**A**) Date palm Biser (unripe) and Tamar (ripe) fruits immediately after harvest, (**B**) naturally ripe Tamar fruits (control), (**C**) unripe Biser fruits, and (**D**) artificially ripe Tamar fruits.

During the years 2019–2020, date palm (cv. Khalas) fruit bunches were collected in August from twelve-year-old trees raised from tissue culture at the Date Palm Research Center of Excellence (DPRC), Training and Research Station, King Faisal University (KFU), KSA (Latitude 25°16′24.4524″ N, Longitude 49°42′28.5948″ E, and Altitude 155 m above sea level). The fruit bunches contained a mixture of ripe (Tamar) and unripe (Biser) fruits (Figure 1A), which were separated into naturally ripe Tamar (Figure 1B) and unripe Biser (Figure 1C) fruits. The fruits were disinfected with Sodium Benzoate solution to minimize the microbial activity. This region represents the major date palm production area of cv. Khalas and has dryland arid climate conditions with very hot dry summers (46–48 °C) and mild winter (7–10 °C). The date palm male flowers bloom from January to March in this region, whereas female flowers bloom from February to April. The harvested Biser sampling fruits were collected from the trees, sorted, cleaned, and stored at 18 °C for initial physical and chemical analyses. Then the date fruits were treated in the treatment chambers of the automated artificial ripening systems with three set-point temperatures (45, 50, and 55 °C) and six set-point RH (30, 35, 40, 45, 50, and 55%). The experiment was designed as a completely randomized design with three replicates. The temperature and RH were maintained in the treatment chambers using respective sensors connected to the computer software for data logging. When the Biser fruits turned brown (ripe) at different time intervals, they were taken to the Fruit Quality Assessment Laboratory, DPRC, KFU, KSA, for physicochemical analysis (Figure 1D).

### 2.2. Construction of the Artificial Ripening System

**Figure 2:**
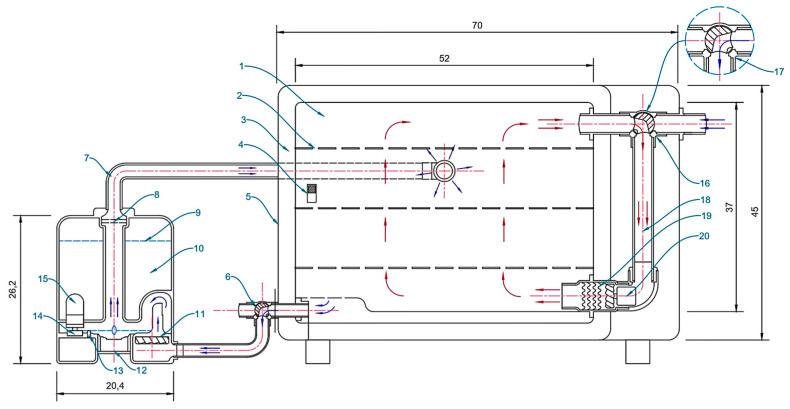
Schematic diagram of an artificial ripening system showing the different parts. (1) Processing chamber, (2) Samples shelves, (3) Glass wool, (4) Temperature & RH Sensor, (5) Outer cover, (6) Electrical 3-Way valve, (7) Mist duct, (8) Plastic mesh, (9) Upper limit of water level, (10) Water tank, (11) DC air fan, (12) Ultrasonic transducer, (13) Water level sensor, (14) Water flow regulator, (15) Filter, (16) Electrical 3-Way valve, (17) The Electrical valve is in the position of air intake from the outside, (18) Air duct, (19) Nickel-chrome heat coil, (20) DC air fan (Dimensions in cm).

**Figure 3:**
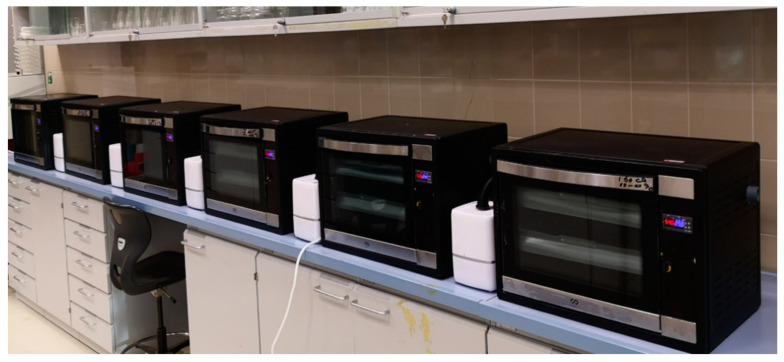
Image of six automated artificial ripening systems.

In this study, eighteen automated laboratory-scale systems for artificial fruit ripening were developed and tested at Date Palm Research Center of Excellence, King Faisal University, KSA, and were used for the experiments of ripening unripe date fruits. Each automated artificial ripening system consisted of four main parts: (1) treatment chamber, (2) heating unit, (3) ultrasonic humidifier, (4) electronic control units (Figure 2). The treatment chamber was a box with 37 cm × 37 cm × 52 cm made from stainless steel with a thickness of 0.1 cm. The chamber is insulated with a layer of glass wool thickness of 0.25 m, and its thermal conductivity is 0.030 at 25 °C. The outer dimensions of the chamber were 45 cm × 45 cm × 70 cm and made from the electrostatic painted galvanized sheet with a thickness of 0.1 cm. All outer surfaces of the ducts also are insulated to reduce heat losses. The inner space of the processing chamber allows the entry of three shelves of the samples made from stainless steel wire mesh with dimensions of 52 cm × 35 cm. The heating unit has consisted of an electrical DC fan and a nickel-chrome heat coil with a maximum power of 500 W. To adjust the RH inside the treatment chamber, we used an ultrasonic humidifier (model: HM3000-B5, Black & Decker, Suzhou, China). The ultrasonic humidifier is developed based on the methods described by the authors in [31,32,33,34] for adjusting RH in the systems. The image of six automated artificial ripening systems is shown in Figure 3.

In this study, we used a simple method based on sensors, an open-source microcontroller board, liquid crystal display (LCD), and Excel to acquire, save, and monitor temperature and RH data inside the treatment chambers of developed systems and weight loss during treatment in real-time. The acquisition data are displayed in the LCD and in an Excel spreadsheet using the PLX-DAQ Excel Macro. The temperature and RH data are obtained through the DHT22 sensors. The weight loss was obtained through the Load Cell sensor with amplifier HX711, then transmitted to the open-source microcontroller of the Arduino UNO board (Microchip ATmega328P, Microchip Technology Inc. W Chandler Blvd, Chandler, AZ, USA).

### 2.3. Physicochemical Characteristics of Date Fruit

Fruit samples were taken at Biser (yellow color) and Tamar stages (brown color, full ripening). Five-kilogram fruits were randomly selected from the five bunches in each palm tree to determine the physicochemical characteristics. These variables were determined according to AOAC standard methods (AOAC, 2005).

#### 2.3.1. Fruit Weight and Size

Fruit weight (g) was measured by Sartorius electronic balance (Sartorius Lab Instruments GmbH & Co. KG, Göttingen, Germany). The fruit length (cm) and diameter (cm) were measured by digital precision vernier caliper (Electron Microscopy Sciences, VWR International, Radnor, PA, USA).

#### 2.3.2. Fruit Firmness

Fruit firmness was measured using a Texture Analyzer (K95590 Digital Penetrometer, Koehler, Bohemia, New York, NY, USA). The texture of all date samples was evaluated with a cylindrical puncture probe with a diameter of 7 mm at room temperature (about 25 °C). The samples with almost similar thicknesses were used to minimize variations between the tested fruit. The traveling speed of the probe and the puncture distance of all tests was 30 mm per minute and 5 mm, respectively. Maximum forces recorded during the punching process were reported as indications of the firmness of the date texture.

#### 2.3.3. Fruit Color

The color parameters of fruit were measured before and after treatments using 100 fruits selected randomly. It was measured using a Hunter lab Color Quest-45/0 LAV color difference meter (Hunter Associates Laboratory Inc., Reston, Virginia, USA) based on the L*, a*, and b* color system. This system is one of the uniform color spaces recommended by the CIE in 1976 to represent perceived color closely. The L* value is the lightness factor that gives values ranging from (0) for black to (100) for white, while a* and b* are chromaticity coordinates. a* value indicates the degree of greenness—redness (ranging from −60 to 0 for green and from 0 to +60 for red), and the b* value indicates the blueness yellowness (ranging from −60 to 0 for blue and from 0 to +60 for yellow). The color difference (ΔE) was determined by the following equation:

$$\Delta E=\sqrt{{\left(L_{2}^{*}-L_{1}^{*}\right)}^{2}+{\left(a_{2}^{*}-a_{1}^{*}\right)}^{2}+{\left(b_{2}^{*}-b_{1}^{*}\right)}^{2}}$$

where, a* is the fruit redness, b* is the fruit yellowness, L* is the fruit lightness, ∆E is the color difference of the fruit before and after treatments.

Effectively the ∆E tolerance value defines an acceptance sphere around the standard or target color. The lower the ∆E value is, the closer the sample is to the standard. For example, the ∆E value of 0.00 means the color of the sample is identical to the color of the standard, and its higher values indicate the variation in color or color difference.

#### 2.3.4. Fruit Moisture Content

Fruit moisture content was determined by putting 10 g fruit samples in an oven (Model ED-260, Binder, Tuttlingen, Germany) at 70 °C until the constant weight. Then moisture content was calculated as gram water per 100 g sample. 

#### 2.3.5. Fruit pH

Ten artificially and naturally ripened date palm fruits from each treatment combination were crushed and a slurry was prepared. The pH of fruit juice extracted from the slurry was measured using a portable pH meter (Model HI-99121, Hanna Instruments, Leighton Buzzard, BedfordshireCity, UK).

#### 2.3.6. Total Soluble Solids

Total soluble solids (TSS) were measured with juice obtained from 10 fruits per treatment by a method modified from Lara et al. [35]. It was determined with a digital refractometer (Model HI-96801, Hanna Instruments, Leighton Buzzard, Bedfordshire, UK), and results were expressed as Brix in juice at 25 °C. An appropriate quantity of each sample was placed on the digital refractometer’s prism plate, and the reading appearing on the screen was directly recorded as total soluble solids.

#### 2.3.7. Fruit Weight Loss

The percentage of fruit weight loss was calculated based on the difference between the weight of the fruit before treatments and after the treatments time. The following equation was used to calculate the weight loss:

$$Wl=\frac{W_{i}-W_{a}}{W_{i}}\times 100$$

where *Wl* is the weight loss (%), *W_i_* is the initial weight of the date sample (g), and *Wa* is the weight of the fruit after treatment (g).

The shrinkage ratio (*S_r_*) for individual fruit was assessed using the following formula:

$$S_{r}=\frac{V_{af}}{V_{ai}}$$

where *V_af_* is the apparent volume after treatment and *V_ai_* is the initial apparent volume.

#### 2.3.8. Sugar Content

Sugars (fructose, glucose, and sucrose) were quantified using a Waters 2010 HPLC system (Waters Corp., Milford, MA, USA) with Empower Software, Shodex Degasser, Autosampler, refractive index detector, and high-performance carbohydrate column (4.6 mm × 250 mm, 4 µm). In addition, silica gel plates were used for TLC (200 µm) and preparative TLC (250 µm) (Analtech Inc., Newark, DE, USA). All solvents used were HPLC grade (Sigma-Aldrich Chemical Company, St. Louis, MO, USA). Date fruits of cv. Khalas were weighed separately and pitted to determine the sugar content. The pulp of date fruits was then cut into small pieces, homogenized with 100 mL water, allowed to remain at room temperature for 1 h, and then centrifuged (10,000 rpm, 15 min). To obtain a total amount of 4 mL, an aliquot (500 μL) of the resultant supernatant was diluted with water (500 μL) and then with acetonitrile (3 mL). Before HPLC analysis, an aliquot (1 mL) of this solution was filtered through a 0.2 μm PTFE membrane [36]. 

Each date fruit’s fructose, glucose, and sucrose sugars were quantified using a carbohydrate column under refractive index detection in HPLC. Using a column heater module, the temperature of the carbohydrate column used for the analyses was kept at 35 °C. The C18 guard column cartridge was replaced, after every 100 injections. Under isocratic conditions, a 25 μL aliquot of standards and test solutions was injected and eluted with a premixed solvent system (water-acetonitrile, 15:85 *v*/*v*) at a flow rate of 1.75 mL min^−1^. The column was equilibrated for 3 min between injections using the same solvent system. Throughout the analyses, the refractive index detector’s attenuation was kept at eight. Fructose, dextrose (glucose monohydrate), and standard sucrose solutions were prepared and analyzed in duplicate using concentrations of 20, 15, 10, 5, and 2.5 mg mL^−1^, respectively. The sugar analyses were carried out three times for all samples. Calibration curves were obtained by plotting the mean peak areas of duplicate runs from three independent experiments against concentrations. The calibration plot for glucose was calculated based on the glucose content in dextrose. Each extract was analyzed in duplicate. The mean peak areas from the duplicate analyses were used to read the concentration of fructose, glucose (reducing sugars), and sucrose (non-reducing sugars) from their respective calibration curves. The data collected were then averaged to determine the quantity of fructose, glucose, and sucrose in each treatment. According to the spectral data, fructose was identified as the mixture of β-D-fructopyranose and β-D-fructofuranose [37], glucose was identified as a mixture of β-D-glucopyranose and α-D-glucopyranose [38], and sucrose was identified as the α-D-glucopyranose, (1→2) β-D-fructofuranose [39].

### 2.4. Statistical Analysis

The recorded data were analyzed statistically using the one-way analysis on variance (ANOVA) technique to assess the significant difference between treatments. The computer software of Statistical Analysis Systems (SAS, 2001) was used for the data analysis. Mean separation was done using the Least Significant Difference (LSD) test after the treatments were found significant at a 5% probability level.

## 3. Results

### 3.1. Performance of the Developed Artificial Ripening Systems

**Figure 4:**
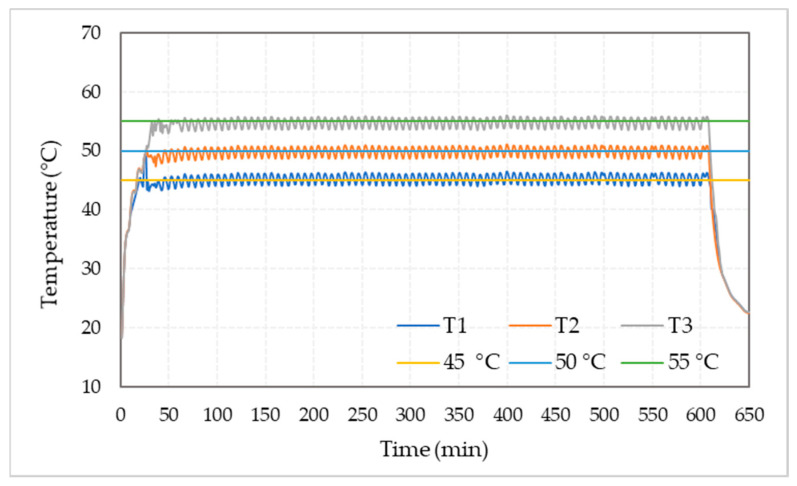
The measured temperatures for the nominal treatment temperature of 45, 50, and 55 °C under 50% target RH.

The measured temperatures using data loggers at the nominal treatment temperature of 45, 50, and 55 °C under 50% target RH are shown in Figure 4. The required delay time for increasing the temperature of the treatment chamber from the initial temperature of 18 °C to the target temperature of 45, 50, and 55  °C were 19, 27, and 39 min, respectively. The measured temperatures values of the treatment chamber were ranged from 0.5 to 1.0 °C higher or lower than the nominal tested temperature.

### 3.2. Physicochemical Characteristics of Date Fruit

#### 3.2.1. Fruit Weight and Size

Date palm fruits ripened naturally had 7.30 g of fruit weight (Table 1). Significantly higher fruit weight was recorded when Biser fruits were artificially ripened at 45 °C + 50 and 55% RH (7.32 g and 7.44 g, respectively), 50 °C + 50% RH (7.32 g), and 55 °C + 45 and 55% RH (7.24 g and 7.17 g, respectively). These treatment combinations were statistically at par with naturally ripened date fruits. Fruit diameter was significantly higher at 50 °C + 50 and 55% RH (21.52 mm each) than other treatment combinations in artificially ripened fruits, whereas it was 19.70 g in naturally ripened fruits. Although there was non-significant effect of different temperatures and RH combinations on fruit length and fruit volume, however, at higher temperatures (50 °C) and RH (55%) combination, both attributes were competitive (33.71 mm and 8.22 mm^3^) to naturally ripened fruits (34.19 mm and 6.99 mm^3^).

#### 3.2.2. Fruit Firmness

Fruit firmness was significantly reduced with an increase in RH at all three temperature gradients, whereas it was maximum at 30% RH at all three set-point temperatures (Table 1). Naturally ripened date fruits had perfect fruit firmness (12.23 N mm^−2^), followed by 45 °C + 50% RH (12.42 N mm^−2^), 50 °C + 50 and 55% RH (12.75 and 11.44 N mm^−2^, respectively), and 55 °C + 55% RH (12.75 N mm^−2^). All these treatment combinations were statistically at par with naturally ripened fruits. Fruit ripened artificially at 45 °C + 55% RH were softer and had 9.16 N mm^−2^ fruit firmness.

#### 3.2.3. Fruit Color 

Chromatic changes of naturally and artificially ripened date palm fruits are assessed by monitoring L*, a*, and b* values (Table 1). In the present study, data regarding fruit color parameters indicated varied values within treatment combinations. Generally, L*, a*, and b* values decreased with increased temperature and RH combinations. Fruit lightness (12.23), a* (9.58), and b* (6.91) values were significantly lowered in naturally ripened fruits. However, higher fruit lightness (42.33) was observed in 45 °C + 30% RH combination, a* (15.69) and b* (29.13) in 50 °C + 30% RH combination. These color parameters were also promising in 50 °C + 50% RH combination.

**Figure 5:**
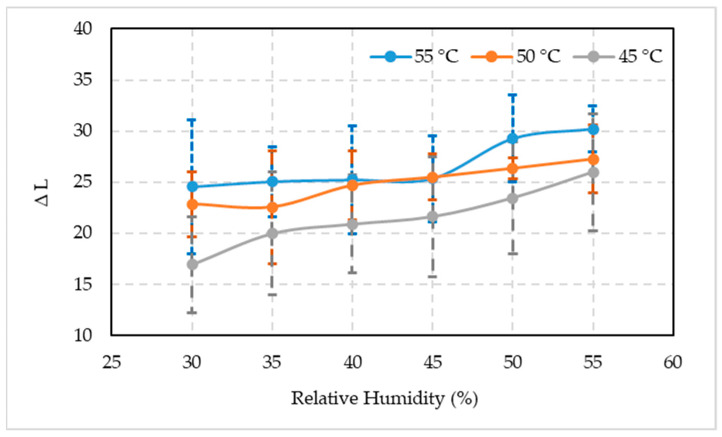
Comparison between the mean values ± standard deviation of the lightness deference (∆L) of artificially ripened date fruits subjected to different temperature and relative humidity combinations.

The surge in the difference in fruit color lightness (∆L) was observed in all three temperatures studied with an increase in RH (Figure 5). These values were minimum when fruits were kept at 30% RH and 45 °C (16.91), 50 °C (22.84), and 55 °C (24.55) temperature regimes. The difference in lightness increased when the RH increased up to 55% at 45 °C (25.96), 50 °C (27.26), and 55 °C (30.17) temperatures. It was closely followed by the fruit placed in 50% RH chambers at 45 °C (23.44), 50 °C (26.34), and 55 °C (29.23) temperatures. Generally, the color of artificially ripened fruits was lighter with the increase in temperature and RH.

**Figure 6:**
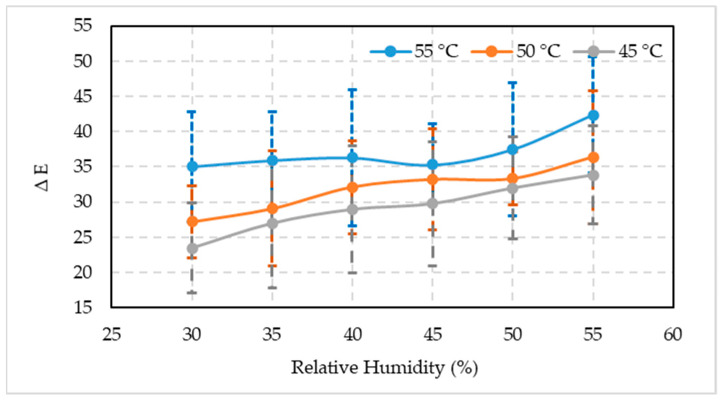
Comparison between the mean values ± standard deviation of the color difference (∆E) of artificially ripened date fruits subjected to different temperature and relative humidity combinations.

The acceptability levels of fruit color variation (∆E) subjected to three temperatures and six RH combinations are shown in Figure 6. A similar trend was observed as it was for the ∆L parameter. A distinct increase in the ∆E was recorded in all three temperature regimes with an increase in RH. Minimum ∆E values were measured when fruits were kept at 30% RM and 45 °C (23.47), 50 °C (27.18), and 55 °C (35.05) temperature regimes. The color difference increased when the RH increased up to 55% at 45 °C (33.83), 50 °C (36.40), and 55 °C (42.38) temperatures, which was followed by the fruits placed in 50% RH chambers at 45 °C (31.98), 50 °C (33.34), and 55 °C (37.49) temperatures. By and large, the color difference of artificially ripened fruits was increased with the increase in temperature and RH.

#### 3.2.4. Fruit Quality 

Artificially ripened date palm fruits at different temperatures and RH showed statistically significant difference (*p* ≤ 0.05) among means regarding fruit moisture content, TSS, pH, fructose, glucose, and total sugars compared to naturally ripened date fruits (Table 2). Higher fruit moisture content (27.17%) was recorded when Biser date palm fruits were artificially ripened at 45 °C + 55% RH. It was also increased at 50 °C (24.47%) and 55 °C (22.90%) temperatures when RH increased to 55%. There was no statistical difference between fruit moisture content when compared to naturally ripened fruits (16.90%) with artificially ripened fruits at 50 °C + 40% RH (16.93%). The artificially ripened fruits showed maximum TSS (71.50 Brix) at higher temperature and RH (55 °C + 55% RH), followed by naturally ripened fruits (70.67 Brix) and 50 °C + 50% RH (68.37 Brix) treatment combination. Fruit pH value of artificially ripened fruits at 50 °C + 50% RH (5.47) combination coincides with the value of naturally ripened fruits (5.48). However, it was much higher at low temperature (45 °C) and higher (50 and 55%) RH. The percentage of fructose were estimated higher (28.50%) in artificially ripened fruits at 55 °C + 55% RH, followed by 50 °C + 55% RH (27.53%). Glucose content was higher (29.50%) at 55 °C + 50% RH, followed by 50 °C + 55% RH (28.47%). Hence, total sugars were higher (57.97%) at 55 °C + 50% RH, followed by 50 °C + 55% RH (56.00%). The values of fructose, glucose and total sugars were estimated 26.30%, 27.67%, and 53.97%, respectively in naturally ripened fruits.

#### 3.2.5. Fruit Weight Loss 

**Figure 7:**
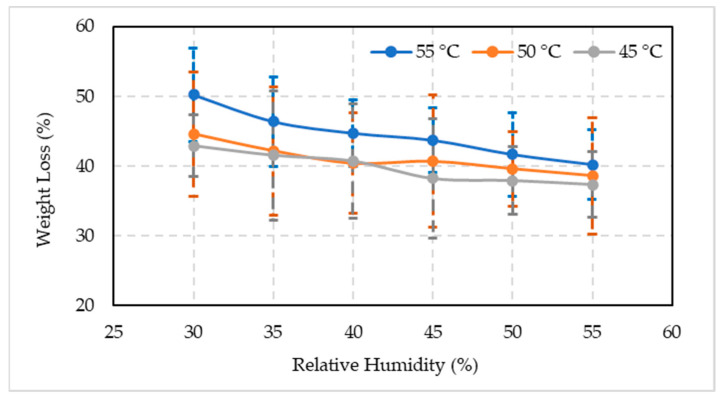
Comparison between the mean values ± standard deviation of the weight loss of artificially ripened date fruits subjected to different temperature and relative humidity combinations.

The results obtained from the present study carried out on artificially ripened fruits at different temperatures and RH showed significant (*p* ≤ 0.05) loss of weight among all the treatment combinations (Figure 7). The general trend was that with the increase in temperature and RH, the weight loss decreased. At high temperature and low RH, the weight loss was high, i.e., loss of fruit weight at 55 °C + 30% RH treatment combination was 50.20%, followed by 50 °C + 30% RH (44.62%), and 45 °C + 30% RH (42.91%) treatment combinations. At the end of study minimum percentage fruit weight loss (40.20%) was observed in 55 °C + 55% RH, followed by 50 °C + 55% RH (38.61%), and 45 °C + 55% RH (37.36%) treatment combinations. Similarly, the treatment combinations of 55, 50, and 45 °C + 50% RH also depicted promising results and had 41.65%, 39.60%, and 37.93% fruit weight loss at 50% RH, respectively.

#### 3.2.6. Fruit Shrinkage Ratio

**Figure 8:**
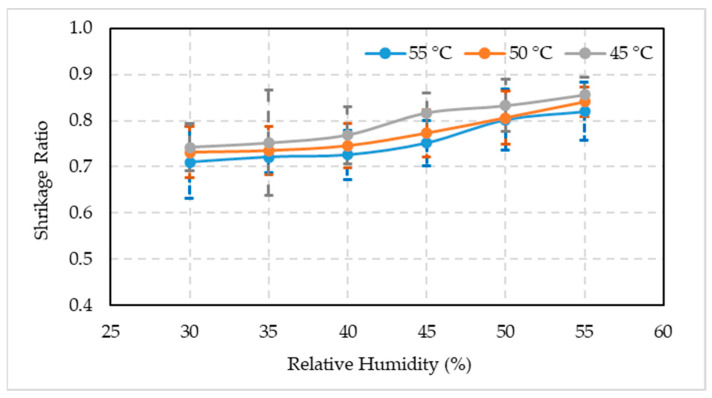
Comparison between the mean values ± standard deviation of the shrinkage ratio of artificially ripened date fruits subjected to different temperature and relative humidity combinations.

The date palm fruit shrinkage ratio (Figure 8) oscillated between 30 and 55% RH at 45 °C (0.74 and 0.86), 50 °C (0.73 and 0.84), and 55 °C (0.71 and 0.82). It indicated that the shrinkage ratio was higher at 45 °C at all RH levels, whereas it decreased at 50 and 55 °C temperatures. The shrinkage ratio trend was similar from 30 to 40% RH at all three temperature regimes. It increased linearly after 45% RH. 

### 3.3. Time Required for Artificial Ripening

**Figure 9:**
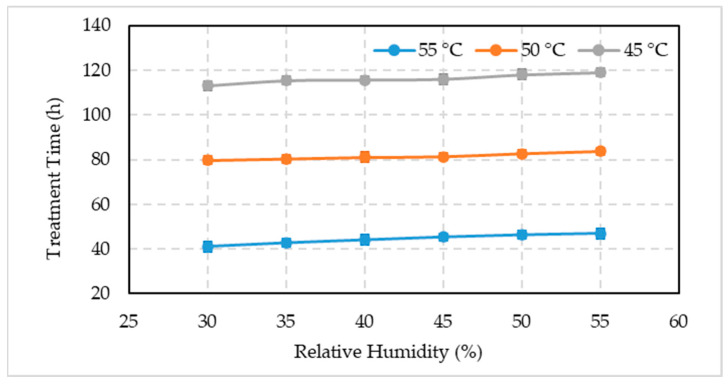
Comparison between the mean values ± standard deviation of the treatment time required for artificial ripening of date fruits under different temperature and relative humidity combinations.

The time required to artificially ripe the unripe Biser fruits varied at different temperatures and RH are shown in Figure 9. The artificial fruit ripening time increased with a decrease in temperature and an increase in RH. However, unripe date fruits placed at 45 °C took 113 h (30% RH) to 119 h (55% RH) to ripe, whereas it took 79.67 h (30% RH) to 83.67 h (55% RH) to ripe at 50 °C. The fruit ripening times oscillated between 41 h (30% RH) to 47 h (55% RH) at 55 °C temperature.

## 4. Discussion

Fruit ripening involves various physiological and biochemical changes that affect the texture, appearance, flavor, nutritional quality, shelf life, and commodity value [10,40,41]. Few previous research studies were conducted on the effects of temperature on ripe date palm fruits (Tamar) for storage, transportation, and postharvest insects and disease control purposes [42,43,44,45]. However, no research was done on unripe Biser fruits from the date palm cv. Khalas to artificially ripen them using different temperatures and RH regimes. The present study revealed the effects of different precised-controlled temperatures and RH to enhance ripening in unripe date palm fruits. Although all temperatures and high RH combinations significantly improve fruit ripening, the 50 °C temperature + 50% RH combination was the most feasible one, enhancing fruit ripening and saving energy compared to 55 °C temperature + 50 or 55% RH combination. 

High temperature reduced fruit size and fruit weight in many fruit plants during ripening. For example, the fruit size and weight of the raspberries [46] and strawberries [47] reduced significantly when the temperature increased in the ripening stage. Fruit weight of artificially ripened fruits increased with increasing temperature in the current study and was comparable to that of naturally ripened fruits. The higher temperature did not significantly affect fruit size [48], which agrees with our studies. Temperature and RH also had a significant effect on fruit firmness. During fruit ripening, specific metabolic and chemical transformations occur in pectin, resulting in textural changes, including firmness. The cell wall and middle lamella polysaccharides play a significant role in fruit texture [49]. Pectin, hemicellulose, and cellulose make up the cell wall polysaccharides, while pectic polysaccharides cross-linked with Ca^2+^ ions make up the middle lamella. Pectin is made up of a backbone in which “smooth” (1→4)-α-D-galacturonan regions with variable degrees of methyl esterification are interrupted by ramified rhamnogalacturonan regions substituted by neutral sugar side chains, such as arabinose and galactose [50]. Thermal exposure produces a significant breakdown of pectic polysaccharides in fruits, resulting in decreased intercellular adhesion and, as a result, fruit softness increased. In fruits, thermal processing causes a pronounced degradation of the pectic polysaccharides, resulting in reduced intercellular adhesion and, consequently, increased softening. The softening of apple tissue was linked to moisture loss and turgor pressure reduction [51]. The results of our study showed that date palm fruits ripened artificially at higher temperatures, and RH reduced the fruit firmness and were similar to the naturally ripened fruits. Due to the increase in RH, moisture content was maintained, and the fruit weight loss was also decreased, which decreased the fruit firmness.

The edible dates go through four ripening stages: Kimri, Khalal (Biser), Rutab, and Tamar [52]. It is fully green at the Kimri stage, whereas in the Khalal (Biser) stage, the fruit color changes from green to yellowish or reddish depending on the cultivar. The color of date fruit tuned to light brown at Rutab stage and dark brown at Tamar stage [4]. Ripening is usually indicated by a dramatic color shift induced by the loss of chlorophyll and the production of certain pigments. One of the most important factors regulating fruit coloring is temperature [53]. Al-Hooti et al. [54] used the CIE technique to study the color of some date palm cultivars. They found that the L* values range from 17.5 to 23.1, indicating that these cultivars ripen to a darker color. In strawberries, high temperature inhibits fruit color pigments [55]. The present study indicated that the fruit color and lightness improved with decreasing temperature and RH. The date fruits, on the other hand, ripened naturally at ambient temperature, and RH had reduced color variables.

The fruit will shrink if the temperature is too high due to moisture content loss [56]. The micro-structure of unripe fruits is affected by artificial ripening at high temperatures, which is reflected in tissue shrinkage as a significant physical change [57]. Fruit structure is altered due to cellular shrinkage, which is linked to water loss at high temperatures. Hills and Remigereau [58] studied the effects of air-drying on parenchyma apple tissue, observed that drying causes the vacuolar compartment to lose water, with little changes in the cytoplasm and cell wall compartments’ water content. Water loss during drying reduces turgor pressure, which is influenced by osmotic pressure and produces plasmolysis [59], affecting cellular wall integrity. The turgor pressure is linked to fruit texture because it maintains the rigidity of cellular tissues. Skin hardening is caused by fruit shrinkage, which can happen during many drying processes. This problem is more common when the drying rate is fast, especially at high temperatures. Internal stresses emerge as the fruit skin dries faster than the core, causing the fruit inside to split and become porous [60]. Non-volatile chemicals move with the diffusing water, precipitate on the fruit surface, and form a crust that maintains the fruit dimensions. As a result, the overall degree of fruit shrinkage is lower at greater drying velocities. Our results indicated a steady increase in fruit shrinkage ratio with the increase in temperature and RH. However, there was a non-significant difference at higher temperatures and RH on the shrinkage ratio.

The results of the present study indicated a rise in fruit moisture content of artificially ripened fruit with increased temperature and RH. The optimal fruit moisture content recorded in naturally ripened fruits was at par with artificially ripened fruits at 50 °C + 40% RH and 55 °C + 45% RH. Enhanced RH increased fruit moisture content and reduced weight loss and firmness. The low RH and high temperature evaporate fruit moisture content, and they start to dry. In general, as the environmental RH increases at a given temperature, the equilibrium moisture content of fruits increases [61]. The TSS and pH of the date palm fruits increased with the rise in temperature and RH, resulting in an increased sensory perception of sweetness [62]. In cherimoya fruit, the TSS increased with increased temperature under storage conditions [63]. A possible explanation for the observed increase in TSS content in the present study could be the increase in sugars with enhanced temperature. The role of soluble sugars in chlorophyll degradation in bananas was suggested by the faster accumulation of high quantities of fructose and glucose in the peel at high temperatures. It accelerated the ripening of banana peel tissue, by faster softening and increased ethylene gene expression [64]. Similarly, when grapes were grown at high temperatures, they contained more sugar [65,66]. A change in sugar content has also been linked to epicarp degreening and regreening in certain citrus fruits [67]. Similar findings were found in the current study, where the sugar content of artificially ripened fruit increased as temperature and RH increased compared to naturally ripened fruits.

Water loss is a physiological process that impacts the key qualitative aspects of fresh fruits, such as saleable weight, appearance, and texture. The present study indicated a decrease in fruit weight loss with increased RH at different temperatures. The loss in fruit weight at high temperature and low RH might be due to a reduction in the moisture content due to the respiration process during the artificial ripening period. Apple cultivars lost weight most rapidly at 20 °C and 30% RH, whereas higher RH reduced apple fruit weight loss [62]. Water stress, caused by a lower than optimum RH in the air surrounding the fruit, can increase the rate of respiration. Weight reduction of more than 5–10% usually result in severe fruit drying, low firmness, shriveling, and poor flavor [68]. Higher RH has a significantly higher effect on fruit weight than at low RH. In a study, evaporation increased by 250% when the RH changed from 98 to 93%, however only by 33% when the RH percentage is changed from 85 to 80% [69]. In the current study, when unripe Biser fruits were subjected to high temperature and low RH combinations, a considerable weight loss was observed in the artificially ripened fruits. The date palm unripe fruits lost more weight when the RH decreased from 40 to 30% than 55 to 50%.

## 5. Conclusions

This study provides information on the individual and combined effects of temperatures and RH on enhancing the artificial ripening of unripe Biser date palm fruits (cv. Khalas) using 18 laboratory-scale controlled systems. The Biser fruits were artificially ripened in these chambers at different temperatures (45, 50, and 55 °C) and RH (30, 35, 40, 45, 50, and 55%). The optimal treatment combination was 50 °C and 50% RH. This treatment combination maintained marketable fruit size, color, firmness, TSS, pH, and sugars. In addition, there was less fruit weight loss and reduced time required for artificial ripening. On the other hand, low temperature (45 °C) and RH (30–45%) delayed the ripening process, deteriorated fruit quality, caused more weight loss, and took more ripening time. Although the high temperature (55 °C) and RH (50–55%) reduced ripening time, but the artificially ripened fruits have higher weight loss. Therefore, it is concluded that artificial ripening of unripe date palm Biser fruits can be achieved using 50 °C temperature and 50% RH combination. Further studies are needed on other commercial date palm cultivars having similar un-consistent in situ fruit ripening issues. Moreover, our controlled environment study results can be evaluated and validated in the field condition using solar energy to reduce costs.

## Figures and Tables

**Table 1:** Comparison of the mean values ± standard deviation of fruit weight (Fw), fruit length (Fl), fruit diameter (Fd), fruit volume (Fv), firmness (F), color lightness (L*), red/green color values (a*), and blue/yellow color values (b*) of naturally ripe fruits (Control) and artificially ripe fruits (ARF) affected by different temperature (T) and relative humidity (RH) combinations.

Ripening Methods	T	RH	Fruit Characteristics							
	°C	%	Fw (g)	Fl (mm)	Fd (mm)	Fv (mm³)	F (N mm^−2^)	L*	a*	b*
Control			7.30 ± 0.3 ^a^	34.19 ± 1.0 ^a^	19.70 ± 1.0 ^ab^	6.99 ± 0.7 ^a^	12.23 ± 0.9 ^hi^	12.23 ± 0.9 ^hi^	9.58 ± 1.5 ^c^	6.91 ± 1.7 ^e^
ARF	45	30	6.58 ± 0.9 ^ab^	30.88 ± 1.3 ^a^	19.58 ± 1.1 ^ab^	6.20 ± 0.7 ^a^	22.24 ± 4.6 ^bd^	42.33 ± 6.1 ^a^	13.51 ± 2.5 ^ac^	26.61 ± 2.8 ^ab^
		35	6.42 ± 1.3 ^ab^	31.27 ± 2.8 ^a^	19.38 ± 1.4 ^b^	6.19 ± 1.1 ^a^	16.35 ± 1.5 ^eh^	40.32 ± 4.2 ^ac^	14.19 ± 3.3 ^ab^	25.06 ± 4.5 ^ac^
		40	6.67 ± 1.2 ^ab^	32.03 ± 1.1 ^a^	20.24 ± 1.1 ^ab^	6.92 ± 0.9 ^a^	15.04 ± 1.1 ^gh^	41.03 ± 6.4 ^ab^	14.22 ± 2.9 ^ab^	23.88 ± 6.6 ^ad^
		45	6.60 ± 1.1 ^ab^	31.21 ± 2.3 ^a^	20.26 ± 1.3 ^ab^	6.77 ± 1.3 ^a^	13.08 ± 3.2 ^gi^	38.61 ± 2.2 ^ad^	13.24 ± 2.1 ^ac^	18.71 ± 6.3 ^ae^
		50	7.32 ± 0.5 ^a^	33.21 ± 1.7 ^a^	19.94 ± 0.8 ^ab^	6.93 ± 08 ^a^	12.42 ± 1.1 ^hi^	32.10 ± 4.5 ^df^	14.07 ± 3.0 ^ab^	17.68 ± 9.4 ^ae^
		55	7.44 ± 0.6 ^a^	33.03 ± 2.4 ^a^	21.12 ± 1.4 ^ab^	7.76 ± 1.4 ^a^	9.16 ± 1.5 ^i^	33.27 ± 4.4 ^ce^	12.58 ± 2.8 ^ac^	13.43 ± 8.0 ^ce^
	50	30	6.43 ± 1.1 ^ab^	32.96 ± 2.2 ^a^	20.90 ± 0.7 ^ab^	7.54 ± 0.8 ^a^	27.14 ± 1.5 ^ab^	35.66 ± 4.0 ^ae^	15.69 ± 1.5 ^a^	29.13 ± 7.5 ^a^
		35	6.83 ± 1.0 ^ab^	32.58 ± 3.7 ^a^	21.19 ± 1.0 ^ab^	7.74 ± 1.6 ^a^	20.27 ± 1.5 ^df^	35.13 ± 5.9 ^ae^	15.49 ± 2.2 ^a^	24.50 ± 6.3 ^ad^
		40	6.63 ± 0.7 ^ab^	31.50 ± 1.3 ^a^	20.13 ± 0.4 ^ab^	6.70 ± 0.4 ^a^	17.99 ± 0.6 ^dg^	35.59 ± 2.5 ^ae^	14.88 ± 2.2 ^ab^	20.52 ± 9.6 ^ad^
		45	7.02 ± 0.8 ^ab^	32.34 ± 2.0 ^a^	20.91 ± 0.8 ^ab^	7.41 ± 0.8 ^a^	13.73 ± 1.0 ^gi^	33.06 ± 4.3 ^cf^	15.09 ± 2.9 ^ab^	20.30 ± 8.8 ^ad^
		50	7.32 ± 1.0 ^a^	33.39 ± 1.8 ^a^	21.52 ± 0.5 ^a^	8.10 ± 0.6 ^a^	12.75 ± 1.0 ^hi^	34.93 ± 5.2 ^ae^	12.78 ± 2.3 ^ac^	15.02 ± 8.1 ^be^
		55	6.90 ± 0.7 ^ab^	33.71 ± 3.2 ^a^	21.52 ± 1.0 ^a^	8.22 ± 1.3 ^a^	11.44 ± 0.6 ^hi^	34.23 ± 4.0 ^be^	14.23 ± 3.3 ^ab^	20.40 ± 9.2 ^ac^
	55	30	5.64 ± 07 ^c^	30.84 ± 2.7 ^a^	19.34 ± 1.1 ^b^	6.08 ± 1.0 ^a^	31.39 ± 1.0 ^a^	31.47 ± 3.1 ^df^	12.46 ± 2.9 ^ac^	11.88 ± 4.9 ^de^
		35	6.49 ± 1.0 ^ab^	32.28 ± 2.4 ^a^	20.49 ± 1.5 ^ab^	7.19 ± 1.5 ^a^	25.51 ± 1.0 ^bc^	32.76 ± 3.5 ^df^	12.56 ± 1.7 ^ac^	13.87 ± 8.6 ^be^
		40	6.48 ± 0.8 ^ab^	31.99 ± 2.7 ^a^	20.20 ± 1.9 ^ab^	6.97 ± 1.9 ^a^	20.93 ± 0.6 ^ce^	32.13 ± 3.7 ^df^	12.66 ± 2.8 ^ac^	12.46 ± 7.9 ^ce^
		45	7.24 ± 0.8 ^a^	33.97 ± 2.7 ^a^	21.07 ± 1.2 ^ab^	7.93 ± 1.3 ^a^	15.70 ± 1.0 ^fh^	31.38 ± 3.5 ^df^	12.54 ± 2.4 ^ac^	11.77 ± 3.3 ^de^
		50	6.99 ± 1.1 ^ab^	32.74 ± 2.9 ^a^	21.07 ± 1.9 ^ab^	7.72 ± 2.1 ^a^	14.72 ± 1.0 ^gh^	25.59 ± 6.1 ^f^	12.46 ± 3.0 ^ac^	13.31 ± 6.0 ^ce^
		55	7.17 ± 0.7 ^a^	33.53 ± 3.6 ^a^	20.57 ± 1.8 ^ab^	7.60 ± 2.1 ^a^	12.75 ± 1.0 ^hi^	29.88 ± 6.8 ^ef^	10.80 ± 3.0 ^bc^	11.99 ± 9.9 ^ce^

The means within each column with the same letter(s) are not significantly different (*p ≤* 0.05).

**Table 2:** Comparison of the mean values ± standard deviation of fruit moisture content (MC), total soluble solids (TSS), fruit pH, fructose content (FC), glucose content (GC), and total sugars (TS) of naturally ripe fruits (Control) and artificially ripe fruits (ARF) affected by different temperatures (T) and relative humidity (RH) combinations.

Ripening Methods	T	RH	Fruit Characteristics					
	°C	%	MC (%)	TSS (Brix)	pH	FC (%)	GC (%)	TS * (%)
Control			16.90 ± 0.7 ^hj^	70.67 ± 5.9 ^ab^	5.48 ± 0.03 ^ce^	26.30 ± 0.1 ^cd^	27.67 ± 0.2 ^bd^	53.97 ± 0.1 ^e^
ARF	45	30	15.50 ± 0.9 ^ij^	62.30 ± 0.9 ^e^	5.80 ± 0.14 ^ac^	24.30 ± 0.5 ^g^	24.87 ± 0.2 ^i^	49.17 ± 0.4 ^i^
		35	18.53 ± 1.1 ^gh^	64.53 ± 1.5 ^de^	5.72 ± 0.26 ^bd^	25.43 ± 0.3 ^df^	24.93 ± 0.8 ^hi^	50.37 ± 0.9 ^hi^
		40	22.47 ± 0.9 ^df^	65.20 ± 0.7 ^ce^	5.92 ± 0.20 ^ab^	25.47 ± 0.4 ^de^	25.93 ± 0.7 ^fi^	51.40 ± 0.5 ^gh^
		45	25.20 ± 0.6 ^ac^	65.50 ± 0.7 ^be^	5.84 ± 0.16 ^ab^	25.83 ± 0.5 ^cd^	26.93 ± 0.2 ^df^	52.77 ± 0.3 ^eg^
		50	26.20 ± 0.8 ^ab^	66.93 ± 0.9 ^ae^	6.07 ± 0.20 ^a^	26.07 ± 0.2 ^cd^	27.63 ± 0.2 ^bd^	53.70 ± 0.4 ^ef^
		55	27.17 ± 0.5 ^a^	66.97 ± 2.0 ^ae^	6.01 ± 0.09 ^ab^	26.57 ± 0.8 ^bc^	27.70 ± 1.0 ^bd^	54.27 ± 1.9 ^de^
	50	30	12.83 ± 0.3 ^kl^	64.23 ± 2.6 ^de^	5.33 ± 0.09 ^e^	24.37 ± 0.4 ^fg^	25.07 ± 0.4 ^gi^	49.43 ± 0.7 ^i^
		35	14.70 ± 0.8 ^jk^	65.03 ± 1.5 ^ce^	5.37 ± 0.01 ^e^	25.60 ± 0.4 ^ce^	26.20 ± 0.2 ^eh^	51.80 ± 0.5 ^fh^
		40	16.93 ± 0.5 ^hj^	66.30 ± 0.2 ^be^	5.38 ± 0.04 ^e^	26.03 ± 0.2 ^cd^	27.07 ± 0.2 ^df^	53.10 ± 0.1 ^eg^
		45	21.57 ± 1.7 ^ef^	67.70 ± 0.6 ^ad^	5.40 ± 0.09 ^de^	26.57 ± 0.2 ^bc^	28.10 ± 0.2 ^bd^	54.67 ± 0.4 ^ce^
		50	23.37 ± 1.2 ^ce^	68.37 ± 0.8 ^ad^	5.47 ± 0.07 ^ce^	27.50 ± 0.4 ^ab^	28.43 ± 0.2 ^ac^	55.93 ± 0.5 ^bd^
		55	24.47 ± 0.9 ^bd^	67.80 ± 0.4 ^ad^	5.41 ± 0.01 ^de^	27.53 ± 0.4 ^ab^	28.47 ± 0.1 ^ac^	56.00 ± 0.5 ^bd^
	55	30	9.43 ± 0.2 ^m^	62.27 ± 0.1 ^e^	5.37 ± 0.01 ^e^	24.57 ± 0.3 ^eg^	25.50 ± 0.3 ^gi^	50.07 ± 0.3 ^hi^
		35	11.07 ± 0.6 ^lm^	64.13 ± 0.6 ^de^	5.37 ± 0.03 ^e^	25.30 ± 0.1 ^dg^	26.27 ± 0.7 ^eg^	51.57 ± 0.8 ^gh^
		40	14.63 ± 0.6 ^jk^	66.57 ± 0.2 ^ae^	5.40 ± 0.02 ^de^	26.27 ± 0.2 ^cd^	27.33 ± 0.3 ^ce^	53.60 ± 0.4 ^ef^
		45	17.40 ± 0.5 ^hi^	70.03 ± 0.2 ^ac^	5.39 ± 0.01 ^de^	27.80 ± 0.1 ^a^	28.77 ± 0.4 ^ab^	56.57 ± 0.5 ^ac^
		50	20.83 ± 0.4 ^fg^	70.47 ± 0.4 ^ab^	5.39 ± 0.01 ^de^	28.30 ± 0.1 ^a^	29.50 ± 0.1 ^a^	57.80 ± 0.2 ^ab^
		55	22.90 ± 0.4 ^ce^	71.50 ± 0.7 ^a^	5.40 ± 0.01 ^de^	28.50 ± 0.4 ^a^	29.47 ± 0.3 ^a^	57.97 ± 0.4 ^a^

The means within each column with the same letter(s) are not significantly different (*p ≤* 0.05). * Sucrose was zero in all treatment combinations, hence, not shown in the above table.
